# Sex Hormones Are Associated With Rumination and Interact With Emotion Regulation Strategy Choice to Predict Negative Affect in Women Following a Sad Mood Induction

**DOI:** 10.3389/fpsyg.2018.00937

**Published:** 2018-06-11

**Authors:** Bronwyn M. Graham, Thomas F. Denson, Justine Barnett, Clare Calderwood, Jessica R. Grisham

**Affiliations:** School of Psychology, The University of New South Wales, Sydney, NSW, Australia

**Keywords:** estradiol, progesterone, emotion regulation, rumination, reappraisal, suppression

## Abstract

Women are particularly vulnerable to anxiety and depressive disorders. This greater vulnerability has been partly attributed to post-pubertal sex hormone fluctuations, estradiol and progesterone, as well as gender-specific tendencies to engage in maladaptive forms of emotion regulation, particularly rumination. To date, no research has investigated whether sex hormones are associated with emotion regulation in women. In the present study, 61 women participated in a sad mood induction task, involving the viewing of an emotive film. Negative affect was assessed immediately and following recovery, along with self-reported use of rumination, reappraisal, and suppression. Serum levels of estradiol and progesterone were assessed through a blood sample taken at the end of the experiment. Regression analyses were used to examine the relationship between serum hormones and self-reported emotional regulation strategy use, and between serum hormones and the impact of these strategies on negative affect. Estradiol levels positively predicted rumination, but not suppression or reappraisal use. Moreover, estradiol and progesterone interacted with emotion regulation strategies to predict negative affect following the sad mood induction. Reappraisal was associated with greater negative affect only in women with high estradiol, and in women with high progesterone. Conversely, rumination was associated with greater negative affect only in women with low estradiol. Together, these results suggest that sex hormone concentration may be an endogenous contextual factor that is associated with the selection and consequences of emotion regulation strategies in women.

## Introduction

Women exhibit an increased incidence, severity, and chronicity of depression, anxiety disorders, and trauma- and stressor-related disorders compared to men (McLean et al., 2011). Post-pubertal fluctuations in sex hormones, such as estradiol (the main estrogen), and progesterone (the main progestogen) may partly contribute to these statistics. Sex differences in these disorders emerge after puberty (Paus et al., 2008), and post-pubertal women commonly report increases in non-pathological anxiety and mood disturbance during the pre-menstrual period, when estradiol and progesterone are declining (Gonda et al., 2008). Similarly, increases in symptom severity amongst women during periods of low or declining estradiol and progesterone occur in depression (Kornstein et al., 2005) panic disorder (Kaspi et al., 1994), obsessive compulsive disorder (Labad et al., 2005), social anxiety disorder (van Veen et al., 2009), posttraumatic stress disorder (Nillni et al., 2015), and schizophrenia (Hallonquist et al., 1993), along with heightened risk of suicide (Saunders and Hawton, 2006).

Sex differences in emotion regulation may also contribute to women’s increased vulnerability to affective disorders (Nolen-Hoeksema, 2012). Emotion regulation refers to a range of strategies used to modify one’s emotional experience and psychopathology, dispositional differences in which are associated with psychopathology (Gross, 2007). For example, suppression of emotional expression tends to increase negative affect, and is associated with anxiety and depressive disorders (Nolen-Hoeksema, 2012). Likewise, rumination, a repetitive pattern of thinking that focuses on the causes, meanings, and consequences of emotional states (Watkins and Moulds, 2005), is a maladaptive strategy that predicts depression (Nolen-Hoeksema, 2000). Conversely, habitual use of reappraisal, which involves thinking about an emotional cue in a way that changes its impact, is associated with enhanced interpersonal functioning and well-being (Gross and John, 2003).

It is well established that women engage in rumination more so than men, a finding which partially accounts for the greater incidence of anxiety and depression amongst women (Butler and Nolen-Hoeksema, 1994). However, studies have indicated that women report to use both maladaptive regulation strategies as well as adaptive strategies, such as reappraisal and acceptance, more so than men (Nolen-Hoeksema and Aldao, 2011; Nolen-Hoeksema, 2012). That is, women report greater use of emotion regulation strategies generally, regardless of their putative helpfulness. Such findings are complemented by research indicating that women have heightened emotional and neural responses (indicated by event related potentials) to moderately negative stimuli, relative to men (Yuan et al., 2009).

Sex differences in emotion regulation have been linked with sex-specific differences in neural activity in the prefrontal cortex (Mak et al., 2009) and event-related potentials (Cai et al., 2016). However, the contextual factors that govern women’s emotion regulation strategy selection, as well as the emotional impact of the selected strategy, in any given moment, remain elusive. It is yet to be investigated whether sex hormone levels at the time of being confronted with an emotional stimulus are associated with the kinds of strategies that women adopt, and the effectiveness of these strategies, in the moment. However, there is evidence that women’s ability to inhibit fearful emotions differs depending on endogenous hormonal state. For example, fear extinction, which is the learned reduction in fear that occurs following repeated exposure to anxiety-eliciting stimuli in the absence of negative outcome, is less effective in women during periods of low estradiol (reviewed in Li and Graham, 2017). The relationship between peripheral progesterone and fear extinction in humans is inconsistent (Li and Graham, 2017), although rodent studies have suggested that progesterone initially augments, and then reverses, estradiol’s facilitation of fear extinction (Graham and Daher, 2016).

Despite procedural differences between fear extinction and cognitive emotion regulation, both depend on a common neural circuitry involving interactions between the ventromedial prefrontal cortex and amygdala (Schiller and Delgado, 2010); a neurocircuitry that is modulated, structurally and functionally, by estradiol and progesterone (Goldstein et al., 2005; van Wingen et al., 2008). It is therefore possible that these hormones may similarly modulate emotion regulation processes. The primary aim of the present study was to determine whether endogenous estradiol is associated with the spontaneous selection of emotion regulation strategies and their emotional consequences in a sample of healthy women. Given the focus of the study was on understanding how sex hormones that fluctuate in women may be related to emotion regulation (rather than examining sex differences in emotion regulation, which has been examined extensively; Nolen-Hoeksema and Aldao, 2011), we limited our investigation to women only. We hypothesized that higher estradiol will predict greater selection of reappraisal and less use of suppression and rumination following a sad mood induction. Furthermore, we hypothesized that lower estradiol will interact with rumination and suppression to predict increased negative affect immediately and post-recovery following a sad mood induction. As noted, progesterone does appear to play a role in both fear extinction and emotion processes, although the nature of this role may be more complex than that of estradiol. Therefore, progesterone was examined as an exploratory predictor in separate models.

## Materials and Methods

### Participants

Sixty-eight female participants were recruited via a public online research participation sign-up system and paid $20 per hour for their participation. Participants were excluded if they were receiving psychological treatment (*n* = 4), or if they reported a score of 19 or above on the Beck Depression Inventory-II (BDI-II; Beck et al., 1996; *n* = 3). The final sample consisted of 61 women, with a mean age of 23.11 years (*SD* = 4.94, range = 18–37).

Power calculations (GPower) indicated that a sample size of 61 women provided adequate power (β = 0.85) to detect a medium effect size at an alpha level of 0.05. Fourteen participants were currently using hormonal contraceptives (mean estradiol 88 pg/ml; mean progesterone 2.22 ng/ml); the remaining participants reported regular menstrual cycles (mean estradiol 315 pg/ml; mean progesterone 7.79 ng/ml). Menstrual cycle stage was not controlled given the objective assessment of serological hormone levels. The decision to include women using hormonal contraceptives, which lead to low endogenous estradiol and progesterone concentrations (Rivera et al., 1999) was made on the basis of their widespread use (around 40% of women in this age range use hormonal contraceptives; Gray and McDonald, 2010). Moreover, women treated with hormonal contraceptives exhibit identical impairments in fear extinction to naturally cycling women with low estradiol and progesterone (Li and Graham, 2017).

### Materials and Measures

#### Questionnaires

The BDI-II assessed current depressive symptoms (Beck et al., 1996) and had excellent internal consistency (Cronbach’s α = 0.81). The suppression and reappraisal items on the Responses to Emotions Questionnaire (REQ; Campbell-Sills et al., 2006) assessed for participants’ spontaneous use of suppression and reappraisal during a negative film clip. A modified version of The Stress Reactive Rumination Scale (SRRS; Robinson and Alloy, 2003) assessed for participants’ spontaneous use of rumination during the film clip. The wording was adjusted to relate to the film clip and irrelevant items were removed, leaving 16 items. A modified version akin to this was used by Moulds et al. (2010). The SSRS includes three subscales assessing both adaptive and maladaptive cognitive tendencies: negative inferential style, hopelessness cognitions and active coping strategies. We summed the two rumination-related subscales (negative inferential style and hopelessness cognitions) to create a composite score, which had acceptable internal consistency (0.74).

#### Mood Rating Scale

A mood rating scale assessed emotional affect at baseline and in response to a negative film clip (Werner-Seidler and Moulds, 2012). Participants rated the current intensity of a range of emotions (‘distracted,’ ‘sad,’ ‘excited,’ ‘bad,’ and ‘happy’) on a 9-point Likert scale that ranged from 1 = *not at all*, to 9 = *very*. A negative mood aggregate score (the average of ‘sad’ and ‘bad’ ratings) was used to gauge changes in negative affect throughout the procedure. Internal consistency was good, ranging from 0.72 to 0.87 across the three time points.

#### Mood Induction Stimuli

Participants viewed an 8-min film clip from either *Dead Poets Society* (Weir, 1989), which shows a suicide scene, or from *My Girl* (Zieff, 1991), which depicts a girl’s response to her best friend’s death. Excerpts from these films have been used to induce sad mood in other studies (Perry et al., 2012; Werner-Seidler and Moulds, 2012).

#### Filler Task

Participants were given 2 min to write a paragraph describing the thoughts and feelings they experienced during the clip, and the strategies they employed to manage these reactions. They were then shown a 5 min slideshow depicting 22 neutral images from the International Affective Picture System (Lang et al., 1997) and asked to rate how pleasant they found each one from 0 (*not pleasant*) to 3 (*very pleasant*). These tasks served as a recovery period to enable the investigation of the persistence of sad mood following the mood induction.

#### Serological Assessment

A venous blood sample was taken approximately 15 min after the experiment at a pathology service located within walking distance from the University by registered nurses trained in venepuncture techniques; all procedures were conducted taking standard infection control precautions. Serum estradiol concentrations were analyzed by Healthscope Pathology Services using an ADVIA Centaur Enhanced Estradiol assay (Siemens), which is a competitive assay that measures serum estradiol concentrations up to 3000 pg/mL (11,010 pmol/L) with a limit of detection of 11.8 pg/mL (43.6 pmol/L). Progesterone levels were analyzed using an ADVIA Centaur Progesterone assay (Siemens), which is a competitive immunoassay that measures serum progesterone concentration up to 60 ng/mL (190.8 nmol/L) with a minimum detectable concentration of 0.21 ng/mL (0.67 nmol/L).

### Procedure

**FIGURE 1:**
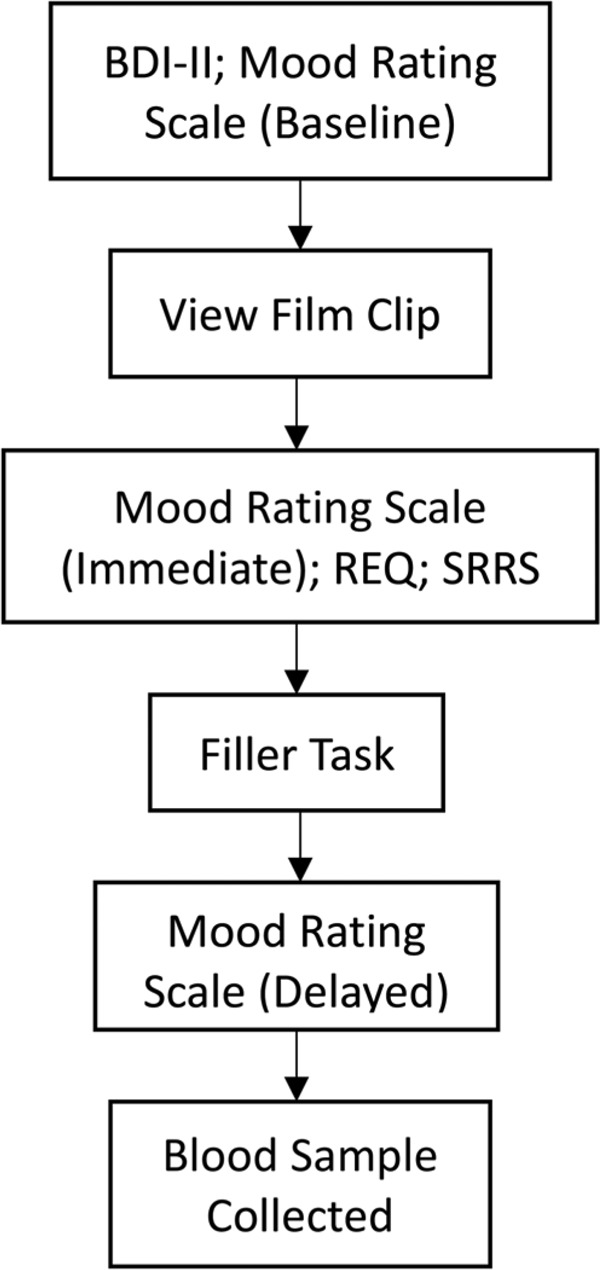
Overview of procedures.

All procedures performed were approved by the UNSW Sydney Human Research Ethics Committee. Written informed consent was obtained from all participants upon arrival in accordance with the Declaration of Helsinki. The protocol was approved by the UNSW Human Research Ethics Committee. A screening interview was conducted to determine participants’ eligibility for the study. Eligible participants completed the BDI-II and the baseline mood rating scale before viewing the film clip. They were instructed to pay close attention to what was happening during the film. At its conclusion, they completed the second mood rating scale, the REQ and the SRRS. They then completed the filler task, followed by the third mood rating scale. Participants were then fully debriefed and accompanied to the local pathology service. See **Figure 1** for an overview of the procedure.

## Results

### Manipulation Check

See **Supplementary Table S1** for individualized raw data. **Table 1** shows the changes in mean negative affect across the experimental phases. To determine whether the film influenced negative affect, a repeated measures analysis of variance was conducted with time (negative affect reported at baseline, immediately post-film, and post-recovery) as the within-subjects factor. There was a significant effect of time [*F*(2,118) = 87.21, *p* < 0.0001] that was primarily quadratic in nature [*F*(1,59) = 122.29, *p* < 0.0001]. Paired-samples *t*-tests confirmed that negative affect at each time point was significantly different to one another, with negative affect at baseline being lower than negative affect at post-film [*t*(59) = -10.49, *p* < 0.0001] and at post-recovery [*t*(59) = -3.59, *p* = 0.001], and negative affect at post-film being greater than negative affect at post-recovery [*t*(60) = 10.22, *p* < 0.0001].

**Table 1:** Mean (±SEM) negative affect ratings across the experimental phases.

	Baseline	Post-film	Post recovery
Mean (±SEM)	1.65 (0.15)	4 (0.23)	2.18 (0.17)

### Predictors of Emotion Regulation Strategy Selection

To determine the predictors of emotion regulation strategy selection, three hierarchical multiple regression analyses (MRAs) were conducted with reappraisal, suppression, and rumination as the dependent variables (**Table 2**). In the first MRA with reappraisal as the dependent variable, negative affect at baseline was entered in Step 1, and suppression, rumination, estradiol, and progesterone were entered in Step 2. Negative affect at baseline did not predict reappraisal scores; however, the second model was significant, and indicated that increased use of suppression was significantly associated with increased use of reappraisal.

**Table 2:** Hierarchical multiple regression analyses predicting strategy use during film.

Strategy	Step	Variable	*R^2^*	Δ*R^2^*	*B*	*SE B*	95% CI	β
Reappraisal	1	Baseline NA	0.05	0.12	0.46	0.27	-0.08, 1.01	0.22
	2	Baseline NA			0.24	0.31	-0.38, 0.86	0.11
		**Suppression**			**0.43**	**0.17**	**0.10, 0.77**	**0**.**35^∗^**
		Rumination			-0.001	0.004	-0.01, 0.01	-0.08
		Estradiol			-0.21	0.51	-1.23, 0.81	-0.07
		Progesterone			-0.004	0.37	-0.75, 0.74	-0.002
Suppression	1	**Baseline NA**	0.15	0.13	**0.67**	**0.21**	**0.25, 1.09**	**0**.**39^∗∗^**
	2	Baseline NA			0.43	0.23	-0.03, 0.89	0.25
		**Reappraisal**			**0.25**	**0.10**	**0.06, 0.45**	**0**.**31^∗^**
		Rumination			0.002	0.003	-0.004, 0.01	0.09
		Estradiol			0.13	0.39	-0.65, 0.91	0.05
		Progesterone			-0.33	0.28	-0.90, 0.23	-0.19
Rumination	1	**Baseline NA**	0.18	0.10	**36.07**	**10.27**	**15.52, 56.62**	**0**.**42^∗∗∗^**
	2	**Baseline NA**			**33.62**	**10.95**	**11.66, 55.58**	**0**.**39^∗∗^**
		Reappraisal			-2.87	5.15	-13.19, 7.46	-0.07
		Suppression			4.61	6.75	-8.91, 18.13	0.09
		**Estradiol**			**40.24**	**18.55**	**3.06, 77.43**	**0**.**34^∗^**
		Progesterone			-5.62	14.12	-0.33.93, 22.69	-0.06

*CI = 95% confidence interval. Bold face font shows relevant significant predictors. Estradiol was log transformed. All continuous predictors were grand mean-centered. ^∗^P < 0.05; ^∗∗^P < 0.01; ^∗∗∗^P < 0.001.*

In the second MRA with suppression as the dependent variable, negative affect at baseline was entered in Step 1, and reappraisal, rumination, estradiol, and progesterone were entered in Step 2. Both models were significant. Negative affect at baseline was a significant predictor at each step, indicating that greater initial negative affect was associated with higher suppression scores. There was no significant change in *R*^2^ for the second model, although reappraisal was a significant predictor. Higher use of reappraisal was associated with greater use of suppression.

In the third MRA with rumination as the dependent variable, negative affect at baseline was entered in Step 1, and reappraisal, suppression, estradiol, and progesterone were entered in Step 2. Negative affect at baseline was a significant predictor at each step, indicating that greater initial negative affect was associated with higher rumination scores. There was a significant change in *R*^2^ for the second model. Higher estradiol levels were significantly associated with greater use of rumination.

We also ran sensitivity analyses with hormonal contraceptive use as a covariate. The only change in significance was that the relationship between estradiol and rumination was reduced to a trend, *p* = 0.056.

### Predictors of Immediate and Delayed Responses to the Sad Mood Induction

To determine whether emotion regulation strategy use differentially predicts immediate and delayed negative affect after the viewing the film depending on estradiol and progesterone levels, we conducted hierarchical linear mixed models using the R 3.3.1 ‘lme4’ package with restricted maximum likelihood estimation (Bates et al., 2015). Because there were two mood ratings provided by each participant (i.e., post-film and post-recovery), we grouped the data by participant. Participant was specified as a random factor and the remaining predictors as fixed factors. The outcome variable was negative affect at both time points. We ran separate models for estradiol and progesterone as predictors because they were strongly correlated in our sample, *r*(59) = 0.67, *p* < 0.0001. We log-transformed both hormones to correct skew, as determined by D’Agostino (1970) skewness tests (estradiol: skew = 1.5549, z = 4.2377, *p* = 2.258e-05; progesterone: 3.2530, *z* = 6.5147, *p* = 7.285e-11). Log-transformation corrected skew for estradiol (skew = 0.44955, *z* = 1.52160, *p* = 0.13) and greatly improved it for progesterone (skew = 1.3928, *z* = 3.9269, *p* = 8.604e-05). We then grand mean centered all predictors, except time, which was coded 0 = post-film, 1 = recovery, and hormonal contraceptive use, 0 = non-users, 1 = users.

In the first block, we entered baseline negative affect. In the second block, we entered hormonal contraceptive use, each emotion regulation strategy, the focal hormone, and time of assessment. In the third block, we added the two-way interactions between time, emotion regulation strategies, and the focal hormone. In the final block we added the three-way interactions between time, emotion regulation strategies, and the focal hormone.

**FIGURE 2:**
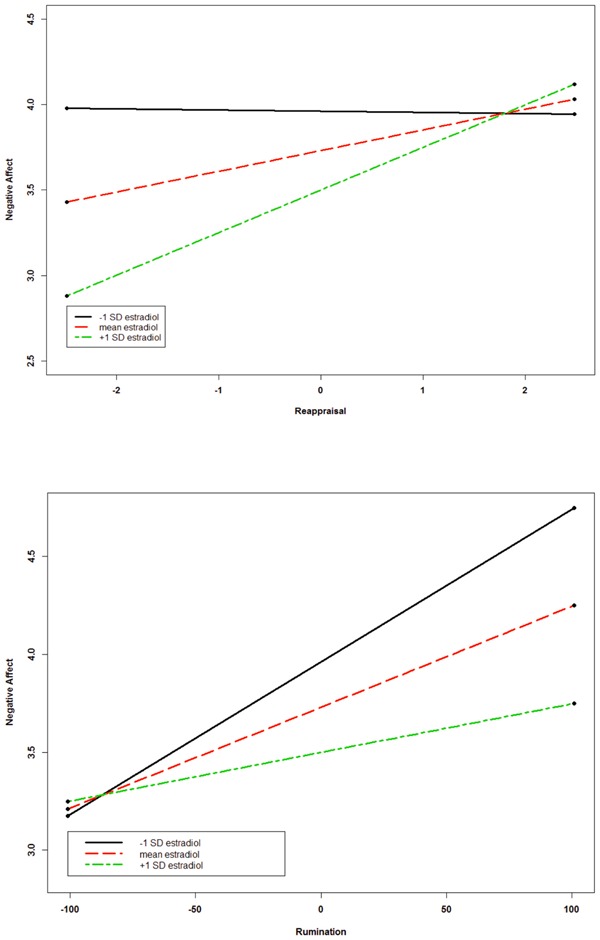
The relationship between reappraisal and negative affect **(top)** and rumination and negative affect **(bottom)** following a sad mood induction as a function of estradiol.

For the estradiol model (**Table 3**), baseline negative affect remained a significant predictor of negative affect in all four blocks. Similarly, the time variable indicated that negative affect was significantly lower at recovery than post-film. In block 2, rumination was positively associated with negative affect. When we entered the two-way interactions at block 3, there was an estradiol × reappraisal interaction and an estradiol × rumination interaction, although they were only marginally significant. **Figure 2** displays these interactions. We plotted values at ± 1 *SD* using Preacher et al. (2006) tools for nested data and simple slopes analyses. There was no relationship between reappraisal and negative affect for women with low or mean levels of estradiol, *p*s > 0.12; however, there was a positive relationship between reappraisal and negative affect for women with relatively high estradiol concentrations, *z* = 2.63, *p* = 0.011. For rumination, there was a positive association between rumination and negative affect, but only for women low in estradiol, *z* = 2.31, *p* = 0.021. At mean levels of estradiol, the relationship between rumination and negative affect approached significance, *z* = 1.80, *p* = 0.072, but was not significant at high levels of estradiol, *p* = 0.43. None of the three-way interactions were significant in the estradiol model.

**Table 3:** Linear mixed model results testing estradiol and emotion regulation predictors of negative affect in response to sad mood induction.

Model	Step	Parameter estimate	CIs	*t*	*P*
***Estradiol Model***					
**Baseline negative affect**	1	**0.62**	**0.35, 0.90**	**4.52**	**<0.001**
**Baseline negative affect**	2	**0.35**	**0.02, 0.68**	**2.16**	**0.036**
Hormonal contraceptive use		-0.52	-1.44, 0.40 ,	-1.13	0.26
**Time**		**-1.83**	**-2.19, -1.47**	**-10.16**	**<0.001**
Suppression		0.02	-0.16, 0.20	0.19	0.85
Reappraisal		0.09	-0.05, 0.23	1.33	0.19
**Rumination**		**0.005**	**0.001, 0.008**	**2.66**	**0.010**
Estradiol		-0.28	-0.75, 0.19	-1.18	0.25
**Baseline negative affect**	3	**0.30**	**-0.01, 0.61**	**1.94**	**0.058**
Hormonal contraceptive use		-0.41	-1.30, 0.48	-0.92	0.36
**Time**		**-1.83**	**-2.19, -1.46**	**-10.11**	**<0.001**
Suppression		-0.02	-0.22, 0.18	-0.21	0.83
Reappraisal		0.12	-0.03, 0.27	1.60	0.12
**Rumination**		**0.005**	**0.001, 0.009**	**2.62**	**0.012**
Estradiol		-0.27	-0.78, 0.24	-1.05	0.30
Estradiol × Time		0.11	-0.33, 0.56	0.51	0.61
Suppression × Time		0.14	-0.06, 0.34	1.41	0.17
Reappraisal × Time		-0.11	-0.27, 0.05	-1.35	0.18
Rumination × Time		0.001	-0.003, 0.005	0.52	0.60
Estradiol × Suppression		-0.15	-0.33, 0.04	-1.57	0.12
**Estradiol × Reappraisal**		**0.15**	**-0.003, 0.30**	**1.63**	**0.055**
**Estradiol × Rumination**		**-0.003**	**-0.01, 0.001**	**-1.71**	**0.094**
**Baseline negative affect**	4	**0.30**	**-0.01, 0.61**	**1.94**	**0.058**
Hormonal contraceptive use		-0.41	-1.30, 0.48	-0.82	0.36
**Time**		**-1.86**	**-2.24, -1.48**	**-9.89**	**<0.001**
Suppression		-0.02	-0.22, 0.18	-0.19	0.85
Reappraisal		0.12	-0.04, 0.27	1.52	0.14
**Rumination**		**0.005**	**0.001, 0.01**	**2.66**	**0.011**
Estradiol		-0.29	-0.80, 0.23	-1.13	0.26
Estradiol × Time		0.15	-0.30, 0.61	0.68	0.50
Suppression × Time		0.14	-0.06, 0.34	1.36	0.18
Reappraisal × Time		-0.10	-0.26, 0.06	-1.20	0.24
Rumination × Time		0.001	-0.003, 0.005	0.42	0.68
Estradiol × Suppression		-0.13	-0.35, 0.09	-1.17	0.25
**Estradiol × Reappraisal**		**0.22**	**0.04, 0.39**	**2.43**	**0.019**
Estradiol × Rumination		-0.003	-0.01, 0.001	-1.39	0.17
Estradiol × Suppression × Time		-0.03	-0.26, 0.20	-0.30	0.76
Estradiol × Reappraisal × Time		-0.13	-0.32, 0.05	-1.44	0.16
Estradiol × Rumination × Time		-0.0003	-0.005, 0.004	-0.14	0.89

*CI = 95% confidence interval. Bold face font shows relevant significant predictors. Estradiol was log transformed. All continuous predictors were grand mean-centered. Time is coded 0 = post-film, 1 = recovery.*

**FIGURE 3:**
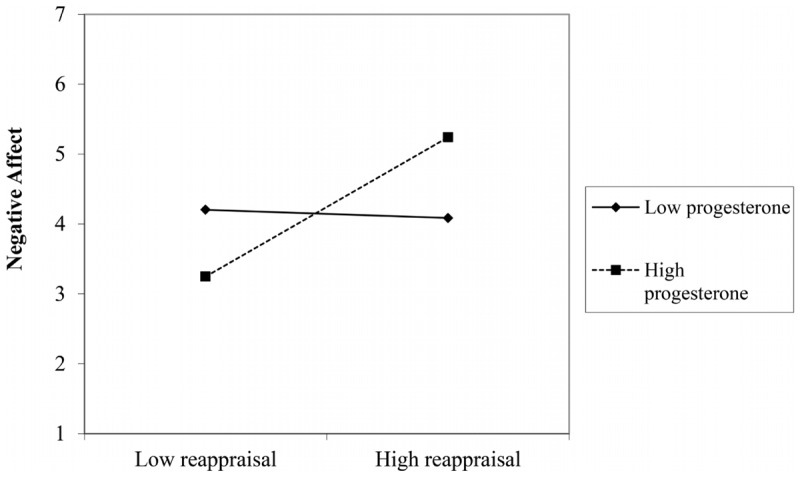
The relationship between reappraisal and negative affect immediately following the film as a function of progesterone.

For the progesterone model (**Table 4**), baseline negative affect and time of assessment remained significant predictors. At block 2, rumination was associated with greater negative affect, but did not retain significance in the later models. There were no significant two-way interactions at block 3, but there was a significant progesterone × reappraisal × time interaction. To probe the nature of the interaction, we conducted separate MRAs for negative affect post-film and post-recovery. There was a significant progesterone × reappraisal interaction immediately following the film, *β* = 0.29, *b* = 0.18, *t*(56) = 2.32, *p* = 0.024, but not at recovery, *β* = 0.06, *b* = 0.03, *t*(56) = 0.45, *p* = 0.66. Simple slopes analyses showed that there was no association between reappraisal and negative affect post-film for women low in progesterone, *b* = -0.02, *t*(56) = -0.19, *p* = 0.85, but there was a significant positive relationship for women high in progesterone, *b* = 0.40, *t*(56) = 3.08, *p* = 0.003 (see **Figure 3**).

**Table 4:** Linear mixed model results testing progesterone and emotion regulation predictors of negative affect in response to sad mood induction.

Model	Step	Parameter estimate	CIs	*t*	*p*
***Progesterone Model***					
**Baseline negative affect**	1	**0.62**	**0.35, 0.62**	**4.52**	**<0.001**
**Baseline negative affect**	2	**0.41**	**0.08, 0.74**	**2.53**	**0.015**
Hormonal contraceptive use		-0.17	-0.95, 0.62	-0.43	0.67
**Time**		**-1.83**	**-2.19, -1.47**	**-10.16**	**<0.001**
Suppression		0.02	-0.16, 0.20	0.24	0.81
Reappraisal		**0.10**	-0.04, 0.24	1.43	0.16
**Rumination**		**0.004**	**0.001, 0.008**	**2.31**	**0.025**
Progesterone		0.07	-0.23, 0.36	0.45	0.65
**Baseline negative affect**	3	**0.41**	**0.07, 0.74**	**2.42**	**0.019**
Hormonal contraceptive use		-0.11	-0.91, 0.68	-0.29	0.76
**Time**		**-1.82**	**-2.19, -1.46**	**-10.12**	**<0.001**
Suppression		-0.06	-0.27, 0.15	-0.59	0.56
**Reappraisal**		**0.16**	**-0.002, 0.32**	**1.98**	**0.053**
Rumination		0.003	-0.001, 0.007	1.55	0.13
Progesterone		0.08	-0.28, 0.44	0.44	0.66
Progesterone × Time		0.11	-0.22, 0.43	0.69	0.51
Suppression × Time		0.15	-0.5, 0.35	1.47	0.15
Reappraisal × Time		-0.11	-0.27, 0.05	-1.36	0.18
Rumination × Time		0.001	-0.003, 0.005	0.62	0.54
Progesterone × Suppression		0.04	-0.12, 0.20	0.44	0.66
Progesterone × Reappraisal		0.07	-0.05, 0.20	1.17	0.25
Progesterone × Rumination		-0.001	-0.005, 0.003	-0.55	0.58
**Baseline negative affect**	4	**0.41**	**0.07, 0.74**	**2.42**	**0.019**
Hormonal contraceptive use		-0.11	-0.91, 0.68	-0.29	0.76
**Time**		**-1.92**	**-2.28, -1.56**	**-10.67**	**<0.001**
Suppression		-0.07	-0.28, 0.14	-0.67	0.51
**Reappraisal**		**0.16**	**0.004, 0.32**	**2.06**	**0.045**
Rumination		0.003	-0.001, 0.007	1.43	0.16
Progesterone		0.12	-0.24, 0.48	0.65	0.52
Progesterone × Time		0.03	-0.31, 0.37	0.18	0.86
Suppression × Time		0.16	-0.03, 0.36	1.68	0.10
Reappraisal × Time		-0.12	-0.27, 0.04	-1.53	0.13
Rumination × Time		0.002	-0.002, 0.005	0.84	0.40
Progesterone × Suppression		0.04	-0.14, 0.23	0.49	0.63
**Progesterone × Reappraisal**		**0.15**	**0.006, 0.30**	**2.09**	**0.040**
Progesterone × Rumination		-0.002	-0.001, 0.003	-0.78	0.44
Progesterone × Suppression × Time		-0.02	-0.19, 0.15	-0.21	0.83
**Progesterone × Reappraisal × Time**		**-0.16**	**-0.30, -0.02**	**-2.24**	**0.030**
Progesterone × Rumination × Time		0.001	-0.003, 0.001	0.61	0.55

*CI = 95% confidence interval. Bold face font shows relevant significant predictors. Progesterone was log transformed. All continuous predictors were grand mean-centered. Time is coded 0 = post-film, 1 = recovery.*

## Discussion

The present study found that, contrary to predictions, women with higher estradiol reported ruminating to a greater extent than those with lower estradiol, whereas estradiol did not predict reappraisal or suppression use. Rumination was the only emotion regulation strategy that predicted negative affect. However, estradiol interacted with both reappraisal and rumination to predict negative affect immediately following the film and at recovery. Specifically, reappraisal was associated with greater negative affect in women with higher, but not lower, estradiol, and rumination was associated with greater negative affect in women with lower, but not higher, estradiol. While progesterone was not associated with emotion regulation strategy use, reappraisal was associated with greater negative affect immediately after viewing the film only in women with higher progesterone, similar to results obtained for estradiol.

Together, our findings suggest that sex hormones may be a potentially important endogenous contextual factor associated with emotion regulation processes in women. As estradiol and progesterone fluctuate widely across the menstrual cycle, we suggest that the effects reported in our study are probably phasic in nature, at least in the case of naturally cycling participants. That is, the tendency to select rumination as a strategy, as well as the impact of rumination and reappraisal on emotional outcomes, may fluctuate with changing estradiol and progesterone levels during the menstrual cycle. Emotion regulation processes may be more stable in women using hormonal contraception (who have relatively unchanging endogenous hormone levels). However, as the present study used a between-persons design, these interpretations must be taken with caution, and future studies using a within-persons design are required to assess whether emotion regulation strategy selection and impact fluctuate in line with the menstrual cycle. In addition, a potential methodological limitation of the current study was the inclusion of both naturally cycling participants, as well as participants using hormonal contraception. Although sensitivity analyses indicated comparable outcomes were obtained when hormonal contraceptive status was included as a co-variate, future studies should assess the relationship between sex hormones and emotion regulation in separate groups of cycling women (whilst controlling for menstrual phase) and women using hormonal contraception. This is particularly important because women using hormonal contraception have low levels of ovarian synthesized hormones, but high levels of synthetic hormones, the impact of which on emotion regulation is currently unknown (Pletzer and Kerschbaum, 2014). Finally, future studies in which a male comparison group is included could shed further light on the hormonal mechanisms that contribute to sex differences in emotion regulation.

The finding that heightened estradiol was associated with greater rumination suggests that sex differences in peripheral estradiol may partly underpin past reports that women engage in rumination to a greater extent than men (Nolen-Hoeksema and Aldao, 2011). This is consistent with the finding that sex differences in rumination emerge at puberty (Jose and Brown, 2008), and raises the possibility that women’s use of rumination may fluctuate with changing estradiol, due to factors including puberty, menstrual cycle phase, hormonal contraception, pregnancy, menopause, etc. Future studies directly comparing rumination levels in men and women during different hormonal states, as well as within-persons examinations of fluctuations in rumination across time are needed to explore this hypothesis more directly.

Although the positive association between estradiol and rumination may seem unexpected, it must be interpreted in light of the finding that the relationship between rumination and mood differed dependent on estradiol. Whereas women with higher estradiol may be more likely to engage in rumination, the association between rumination and negative affect was only apparent in women with lower estradiol. These findings are broadly consistent with an emerging consensus that periods of heightened estradiol may be protective, whereas periods of low estradiol may be associated with increased vulnerability to the development and expression of anxious and depressive symptoms (Kornstein et al., 2005; Nillni et al., 2015). Our findings add to this literature by suggesting that the detrimental impact of rumination may be exacerbated during periods of lower estradiol, and thus may contribute to cyclic increases in psychiatric symptom severity amongst women, a possibility that requires further investigation in clinical populations using a within-persons design.

A surprising outcome was that suppression and reappraisal use were positively related, which is inconsistent with literature demonstrating the absence of a relationship between suppression and reappraisal (Gross and John, 2003). However, past literature has predominantly focused on retrospective assessments of trait reappraisal and suppression, and has rarely examined “in the moment” strategy selection, which is likely to be influenced by contextual factors (Bonanno and Diminich, 2013). The positive relationship between reappraisal and suppression in the present all female cohort is consistent with findings that women report using most emotion regulation strategies (i.e., both adaptive and maladaptive) to a greater extent than men (Nolen-Hoeksema and Aldao, 2011). Thus, when women engage in effortful emotion regulation, they may sample multiple methods, including both suppression and reappraisal.

An equally surprising finding was that in women with higher estradiol, and in women with higher progesterone, reappraisal was associated with greater negative affect, which is inconsistent with literature suggesting that reappraisal is associated with better emotional outcomes (Gross, 2007). It may be the case that, compared to women with lower estradiol and progesterone, those with higher estradiol and progesterone who were most affected by the film were more motivated to use reappraisal to prevent the further exacerbation of their negative mood that may result from dwelling on the film. As reappraisal use and mood at post-film were assessed concurrently, the time course of the effects cannot be discerned. Relatedly, as participants were not instructed to engage in specific emotion regulation strategies, claims cannot be made regarding the causal effects of emotion regulation strategy selection. However, in keeping with this suggestion, there was no relationship between reappraisal and rumination, and further, the relationship between reappraisal and negative affect was observed immediately after the film, but not following a recovery period, at least in the case of women with progesterone. Irrespective of the exact explanation, the fact that reappraisal was differentially associated with negative affect depending on hormonal levels is consistent with the finding that the impact of rumination was also different depending on estradiol levels. Future studies in which estradiol and progesterone are manipulated are required to determine the causal influence of these hormones on emotion regulation strategy selection, and the impact of these strategies. In addition, as hormones were assayed at the end of the experimental procedure, it is unknown whether viewing the films, and/or engaging in emotion regulation altered hormone levels differentially amongst participants. As other experimental paradigms have been demonstrated to rapidly alter sex hormones (Edelstein et al., 2012) future studies should assay hormones both prior to and after engagement in emotion regulation.

The mechanisms that might account for hormonal-associated variations in the impact of reappraisal and rumination on mood are unclear and warrant further investigation. Nonetheless, if it is the case that the influence of emotion regulation strategies on mood varies with estradiol and progesterone, then this raises several hypothesized pathways by which hormonal levels might account for sex differences in patterns of emotion regulation use and psychopathology in women compared to men. For example, these findings suggest that the efficacy of a given emotion regulation strategy in regulating mood may be less consistent in women than men, due to the greater fluctuations in sex hormones amongst women. Such a possibility is consistent with our recent report that the effectiveness of cognitive restructuring in inhibiting conditioned fear responses is significantly reduced during periods of lower endogenous estradiol (Graham et al., 2017). Indeed, this may account for why women engage in a multitude of strategies when confronted with a negative stimulus, as each of which may have had varying success in influencing emotions in the past. Moreover, the possible instability in the consequences of emotion regulation due to changing hormonal states may decrease the perceived (or actual) controllability of negative emotions, which is a key factor in the development of anxiety and depressive disorders (Gallagher et al., 2014).

Finally, if there are periodic changes in the consequences of emotion regulation strategies on mood due to hormonal fluctuations, then this may have significant implications for the implementation of cognitive therapy for mood and anxiety disorders in women. As cognitive therapy depends on the engagement of reappraisal-type strategies and the elimination of strategies like rumination (Barlow et al., 2004), the efficacy of cognitive therapy in women may be influenced by changes in hormonal state. Although these hypotheses are at this stage speculative, the present findings suggest that taking hormonal status into consideration in future studies of emotion regulation may lead to promising new avenues for understanding the basis of women’s greater vulnerability to psychopathology.

## Author Contributions

BG, JB, CC, and JG contributed conception and design of the study. JB and CC performed data collection and organized the database. BG, TD, and JG performed the statistical analysis. BG wrote the first draft of the manuscript. TD wrote sections of the manuscript. All authors contributed to manuscript revision, read and approved the submitted version.

## Conflict of Interest Statement

The authors declare that the research was conducted in the absence of any commercial or financial relationships that could be construed as a potential conflict of interest.

## Supplementary Material

Table S1 Raw data for each participant.
